# Efficacy of low-frequency repetitive transcranial magnetic stimulation combined with risperidone in the treatment of schizophrenia patients with auditory verbal hallucinations

**DOI:** 10.12669/pjms.41.11.13005

**Published:** 2025-11

**Authors:** Wanyun Zou, Ce Chen, Ziye Huang, Xinwu Ye, Xiaozhuang Jin, Haoran Chen

**Affiliations:** 1 Wanyun Zou Department of Geriatric Psychiatry, Wenzhou Seventh People’s Hospital, Wenzhou, Zhejiang Province 325000, P.R. China; 2 Ce Chen Department of General Psychiatry, Wenzhou Seventh People’s Hospital, Wenzhou, Zhejiang Province 325000, P.R. China; 3 Ziye Huang Department of General Psychiatry, Wenzhou Seventh People’s Hospital, Wenzhou, Zhejiang Province 325000, P.R. China; 4 Xinwu Ye Department of Geriatric Psychiatry, Wenzhou Seventh People’s Hospital, Wenzhou, Zhejiang Province 325000, P.R. China; 5 Xiaozhuang Jin Department of General Psychiatry, Wenzhou Seventh People’s Hospital, Wenzhou, Zhejiang Province 325000, P.R. China; 6 Haoran Chen Department of General Psychiatry, Wenzhou Seventh People’s Hospital, Wenzhou, Zhejiang Province 325000, P.R. China

**Keywords:** Auditory verbal hallucinations, Low frequency, Repetitive transcranial magnetic stimulation, Risperidone, Schizophrenia

## Abstract

**Objective:**

This study aimed to determine the efficacy and safety of low-frequency (1 Hz) repetitive transcranial magnetic stimulation (rTMS) combined with risperidone in the treatment of schizophrenia patients with auditory verbal hallucinations (AVH).

**Methodology:**

This retrospective cohort study was conducted in Wenzhou Seventh People’s Hospital and included data from 100 schizophrenia patients with AVH, treated from May 2022 to May 2024. Among them, 50 patients who received low-frequency rTMS combined with risperidone were matched with the cohort of patients who received risperidone alone in a ratio of 1:1. The scores of the Psychotic Symptom Rating Scale (PSYRATS), Auditory Hallucination Rating Scale (AHRS) and Positive and Negative Syndrome Scale (PANNS) were compared between the two groups at baseline, at the end of treatment (five weeks) and after one month of follow-up.

**Results:**

PSYRATS, AHRS, and PANSS scores of the two groups decreased with time (p<0.05). After treatment, the general psychopathological symptoms, positive symptom scores, and PANSS total scores of the rTMS/risperidone group were lower than those of the risperidone group (p < 0.05). There was no difference between the two groups at one-month follow-up (p > 0.05). The incidence of adverse reactions during treatment was comparable in the two groups (p > 0.05).

**Conclusions:**

The results of this study showed that risperidone, combined with 1Hz rTMS, showed no benefit in improving the auditory hallucination symptoms of schizophrenia patients with AVH. However, the combined regimen could significantly improve the positive symptom scores of patients during treatment.

## INTRODUCTION

Auditory verbal hallucinations (AVH) occur in approximately 60–70% of patients with schizophrenia and are strongly associated with an elevated risk of suicide and aggressive behavior.^1^,^2^ Currently, therapeutic strategies for AVH primarily include psychotherapy, neuromodulatory interventions, and pharmacological treatments with antipsychotic agents such as clozapine, olanzapine, and risperidone.^2^,^3^ Risperidone is a second-generation antipsychotic that alleviates auditory hallucinations by blocking the synthesis of dopamine D2 receptors and other neurotransmitters in the central nervous system.^3^,^4^ Compared with clozapine, risperidone has been reported to exert greater effects on preserving the integrity of white matter structural networks.^5^ Nevertheless, approximately 20–40% of patients treated with risperidone exhibit poor tolerability or an insufficient therapeutic response.^6^,^7^ Cognitive behavioral therapy (CBT) is the most studied psychological intervention for AVH.^8^,^9^

The main goal of CBT is not to reduce the incidence of AVH, but to alleviate the negative reactions and adverse beliefs associated with the hallucinations.^8^ However, CBT often requires a long course of treatment, and its effectiveness is impacted by many factors such as patient compliance and the skills of the psychotherapist.^9^ Among neuromodulatory approaches, repetitive transcranial magnetic stimulation (rTMS), a non-invasive brain stimulation technique, has been extensively investigated as a potential intervention for AVH.^10^,^11^ Neuroimaging and neurophysiological studies have shown that the occurrence of AVH is closely related to the abnormal excitation of the brain language network, especially the overexcitation of neurons in the left superior temporal gyrus (STG), left inferior frontal gyrus (IFG), and other regions.^10^,^12^ In the treatment of auditory hallucination, low-frequency rTMS was shown to have an inhibitory effect on cortical neurons.^11^

However, the therapeutic efficacy of low-frequency rTMS for AVH remains inconclusive, with inconsistent findings across clinical trials.^13^-^15^ A recent meta-analysis showed that low frequency (<5 Hz) rTMS led to multidimensional changes in brain activity and structure of patients with refractory AVH, which somewhat improved their symptoms.^13^ However, other studies have failed to demonstrate the efficacy of rTMS in patients treated for three weeks or those with drug-resistant AVH.^14^,^15^ Such differences may be related to different factors such as the population, sample size, treatment parameters (such as age, gender, stimulation site, intensity, frequency, course of treatment, etc.) in the study.^13^-^15^

This study aimed to assess the efficacy and safety of low-frequency rTMS as an adjunct to risperidone in the treatment of patients with schizophrenia and AVH.

## METHODOLOGY

This retrospective cohort study included data from one hundred schizophrenia patients with AVH, treated in Wenzhou Seventh People’s Hospital from May 2022 to May 2024. Among them, 50 patients who received a low-frequency rTMS combined with risperidone were matched with the cohort who received risperidone alone in a ratio of 1:1. The matching conditions were age, gender, course of disease, educational level, body mass index (BMI), type of disease, and marital status.

### Ethical approval:

This study has been approved by the Ethics Review Committee of Wenzhou Seventh People’s Hospital (EC-20200610-02).Date June 09, 2020

### Inclusion criteria:

- Meets the diagnosis of schizophrenia or schizoaffective disorder, and the criteria of true auditory hallucination according to the Chinese Classification of Mental Disorders (CCMD-3).
- The total score of the Positive and Negative Syndrome Scale (PANNS) of 60 or more.
- Severe daily refractory auditory language hallucinations despite the effective dose and duration of antipsychotic drugs (except risperidone).
- Age 18-65 years old.
- The treatment lasted for six weeks, and the patients were followed up for more than one month.

### Exclusion criteria:

- Patients with a personal or family history of seizures.
- A history of traumatic brain injury or brain disease.
- Patients with severe behavior disorders.
- Current drug abuse patients.
- Pregnant patients.
- Patients with mental retardation.

### Treatment regimens:

### Risperidone treatment:

Patients received oral risperidone (Xi’an Janssen Pharmaceutical Co., Ltd., China; specification: 1mg/tablet). The initial dose was 1mg/d. The dose was increased by 1-mg every 1-2 days. The treatment dose was adjusted to 4-6 mg within two weeks, and the maximum daily dose was ≤ 6 mg. The treatment lasted for six weeks.

### rTMS:

In addition to risperidone treatment, patients received low-frequency rTMS treatment using the Magstim Rapid System, equipped with eight-digit coils (Yiguide YCD-I, Wuhan, China). The parameters were as follows: frequency, One Hz; stimulus intensity, set at 110% of the individual’s resting motor threshold (RMT) for the left hemisphere, with 1200 pulses/day. The left temporal parietal junction (TPJ) was stimulated with 80% motor threshold, and every 20 treatment sequences were composed of one intervention, continuous stimulation for five seconds, interval of 30 seconds, and 800 stimuli/treatment; 20 minutes/time, five times/week.

### Collected data:

The following indices were collected from all patients:

- Demographic characteristics, including age, gender, course of disease, education level, BMI, disease type, and marital status.
- Psychotic Symptom Rating Scale (PSYRATS), Auditory Hallucination Rating Scale (AHRS), and PANSS scores at different time points. PSYRATS included the auditory hallucination subscale (four items) and the delusion subscale (three items), with the higher score (0-4 points) indicating more serious symptoms. The AHRS scale included four aspects: voice content, frequency, loudness, and patient pain. The higher the score, the more serious the patient’s auditory hallucination symptoms. PANSS included positive symptom scale (seven items), negative symptom scale (seven items) and general psychopathological symptom scale (16 items), with 1-7 points for each item. The higher score was associated with more serious symptoms.
- Adverse events, including pain, tachycardia, dizziness, drowsiness, nausea, and vomiting.

### Statistical analysis:

The collected data were entered into the Excel spreadsheet and analyzed using the SPSS/PC statistical software (version 25.0; IBM Corp, Armonk, NY, USA). The counting data were expressed as n (%), and the differences between groups were tested using the chi-square test. Visual (histogram and probability map) and analytical (Kolmogorov-Smirnov/Shapiro-Wilk tests) methods were used to evaluate whether the variables follow a normal distribution. The measurement data, which followed a normal distribution, were expressed as mean ± standard deviation (SD). The differences between the two groups were tested using an independent sample t-test. The data with non-normal distributions were represented by the median and interquartile range (IQR); the Mann-Whitney U test was used for comparison between groups. The Wilcoxon signed-rank test was used to compare the results before and after treatment. The statistical significance was set at p < 0.05. Prism 9.0 software (Graphpad, San Diego, USA) was used to draw PSYRATS, AHRS and PANSS score change charts in different periods.

## RESULTS

**Fig.1:**
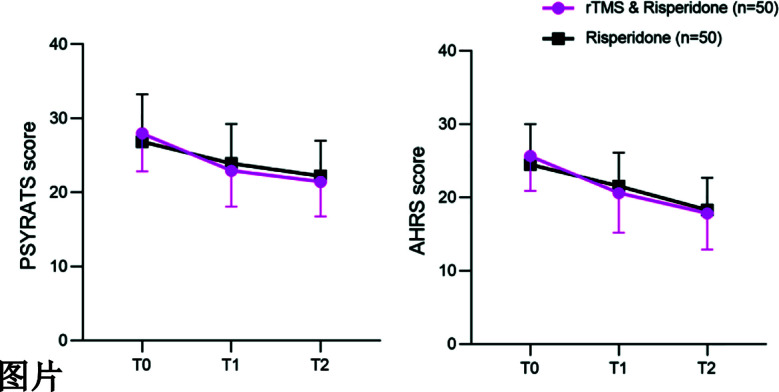
Change curves of PSYRATS and AHRS scores at different periods. T0: At baseline; T1: After the last treatment; T2: Follow-up for one month. *P<0.05 compared to Risperidone group.

This study included data from 100 patients with AVH, 66 males (60.0%) and 34 females (34.0%). The average age was 43.2 ± 11.7 years (range, 19-65 years). There were 50 cases in the risperidone group and 50 cases in the rTMS and risperidone group, with a 1:1 matching ratio. There was no significant difference in the basic characteristics between the two groups (P>0.05) (Table-I). The PSYRATS and AHRS scores of both groups decreased over time (P<0.05), with no significant intergroup difference (P>0.05) (Fig.1).

**Table-I:** comparison of basic characteristics between the two groups.

Variables	rTMS & Risperidone (n=50)	Risperidone (n=50)	t/χ^2^/Z	P
Age (years), mean±SD	41.7±10.5	44.7±12.6	-1.280	0.203
Sex, n(%)			0.694	0.405
Male	34 (68.0)	30 (60.0)		
Female	16 (32.0)	20 (40.0)		
Course of disease (years), M(IQR)	13 (9-16)	12 (9-26)	-0.712	0.477
Educational level, n(%)			1.974	0.160
Junior high school and below	35 (70.0)	41 (82.0)		
High school and above	15 (30.0)	9 (18.0)		
BMI (kg/m²), mean±SD	24.8±3.0	25.7±3.1	-1.561	0.122
Primary diagnostics, n(%)			0.198	0.656
Schizophrenia	37 (74.0)	35 (70.0)		
Schizoaffective disorder	13 (26.0)	15 (30.0)		
Marital status (single), n(%)	37 (74.0)	49 (78.0)	0.219	0.640

**Fig.2:**
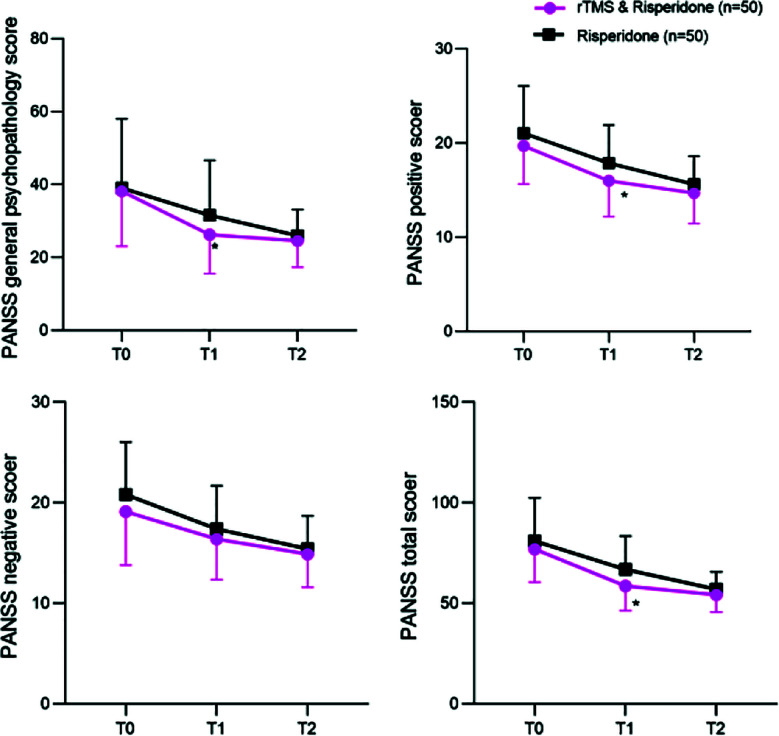
The change curves of PANSS scores and total scores for two groups at different time periods; T0: At baseline; T1: After the last treatment; T2: Follow up for one month. *P<0.05 compared to Risperidone group.

The scores of each sub-item and the total score of PANSS in both groups decreased over time. Patients in the rTMS and risperidone group had lower general psychopathology, positive symptoms, and total score than the patients in the risperidone group after the last treatment (P<0.05). There was no significant difference in scores between the two groups at one month of follow-up (P > 0.05) (Fig.2). Table-II shows the median and interquartile range of PANSS scores and total scores for the two groups at different time periods. As shown in Table-III, there was no significant difference in the incidence of overall and specific adverse reactions between the two groups (P>0.05).

**Table-II:** PANSS scores and total scores for two groups at different time periods.

Variables	rTMS & Risperidone (n=50)	Risperidone (n=50)	Z	P
***T0***				
General psychopathology	34 (27-46)	33 (26-39)	-0.159	0.874
Positive symptoms	19.5 (17-22)	20 (17-25)	-1.156	0.247
negative symptoms	17 (15-22)	19 (17-25)	-1.919	0.055
Total score	73 (63-88)	71.5 (65-90)	-0.731	0.465
***T1***				
General psychopathology	22 (20-30)	26 (22-35)	-2.053	0.040
Positive symptoms	15.5 (13-19)	17 (14-20)	-2.248	0.025
negative symptoms	15 (14-18)	16 (15-20)	-1.490	0.136
Total score	55.5 (50-64)	60 (56-75)	-2.867	0.004
***T2***				
General psychopathology	22.5 (20-29)	26 (20-32)	-1.094	0.274
Positive symptoms	14 (13-16)	15 (14-17)	-1.904	0.057
negative symptoms	14 (13-16)	15 (14-16)	-1.346	0.178
Total score	51 (48-60)	57 (50-62)	-1.881	0.060

T0: At baseline; T1: After the last treatment; T2: Follow-up for one month.

**Table-III:** Comparison of Adverse Reactions between Two Groups.

Adverse reactions	rTMS & Risperidone (n=50)	Risperidone (n=50)	χ^2^	P
Pain	12 (24.0)	6 (12.0)	2.439	0.118
tachycardia	9 (18.0)	5 (10.0)	1.329	0.249
Dizziness	13 (26.0)	7 (14.0)	2.250	0.134
Drowsiness	6 (12.0)	3 (6.0)	1.099	0.295
Nausea and vomiting	7 (14.0)	5 (10.0)	0.379	0.538

## DISCUSSION

This study aimed to investigate whether low-frequency rTMS can enhance the efficacy of risperidone in treating AVH. The results showed that, after six weeks of treatment and at one month of follow-up, there was no significant difference in PSYRATS and AHRS scores between the two patient groups. This result indicates that the combination of rTMS did not yield higher benefits in improving auditory hallucinations in AVH patients.

The results of this study differ from related studies. Brunelin et al.^16^ found that low-frequency 1 Hz rTMS, administered 30 times over three weeks, can significantly reduce the AHRS score of patients with AVH. In this study, the treatment frequency of rTMS was five times per week, with 30 sessions within six weeks. The selection of 1 Hz frequency was based on both clinical practice and neurophysiological evidence. Low-frequency (≤1 Hz) rTMS has been shown to exert an inhibitory effect on cortical excitability. In patients with AVH, neuroimaging studies have demonstrated abnormal hyperactivation of the left temporoparietal junction (TPJ), a region critically involved in auditory language processing and inner speech regulation. Applying 1 Hz stimulation to this area is therefore expected to suppress excessive neuronal activity and help restore the balance of the auditory-linguistic network. Previous studies have reported clinical benefits of 1 Hz rTMS in reducing AVH severity through this mechanism.^17^,^18^ The left temporoparietal junction (TPJ) was chosen as the stimulation site because neuroimaging studies consistently demonstrate abnormal hyperactivation of this region in patients with auditory verbal hallucinations.^19^ The TPJ plays a crucial role in auditory language processing and self-monitoring of inner speech, and low-frequency rTMS applied to this area has been shown in prior studies to reduce AVH severity.^20^ Nevertheless, cortical excitability and functional anatomy vary considerably across individuals. In our study, coil positioning relied on conventional anatomical landmarks rather than individualized brain mapping, which may have reduced targeting precision. Future research should therefore employ imaging-guided neuronavigation or functional brain mapping to optimize accuracy, and should also explore alternative stimulation targets such as the left superior temporal gyrus or the inferior frontal gyrus. It is possible that due to the longer interval between low-density stimuli, the Long-Term Inhibition (LTD) effect may partially dissipate, leading to a decrease in the cumulative efficiency of inhibition and difficulty in forming sustained excitatory regulation of brain regions.^16^ Yang et al.^21^ found that compared with risperidone combined with low-frequency rTMS pseudo stimulation therapy, actual stimulation therapy for AVH can significantly improve auditory hallucination symptoms and cognitive function. It is plausible that the discrepancy may be due to the higher average age of the population in the current study (43.2 ± 11.7 vs. 32.1 ± 8.0 years in^21^). Studies have shown that women and young adults who receive rTMS treatment have better outcomes.^22^ This effect may be related to the degree of cortical atrophy, which increases the distance between the coil and the target brain area.

Despite using a comparable treatment course, our study did not observe significant improvement in AVH symptoms with the combined therapy, which contrasts with some previous reports. Several factors may account for this discrepancy. First, the stimulation density in our protocol, although spanning six weeks, may have been lower than that of more intensive or accelerated regimens, potentially leading to insufficient cumulative inhibitory effects. Second, the mean age of our cohort was relatively high, and many patients had long disease durations and histories of treatment resistance, which may have reduced responsiveness to both risperidone and rTMS. Third, coil positioning was performed using conventional anatomical landmarks rather than imaging-guided neuronavigation, which could have limited the accuracy of targeting hyperactive TPJ subregions. Taken together, these factors suggest that patient heterogeneity and treatment resistance play a crucial role in treatment outcomes. Future research should therefore employ individualized, imaging-guided protocols and explore optimized stimulation parameters to better address refractory AVH.

Previous research showed that the likelihood of cortical atrophy is age-related, with younger patients exhibiting lower incidence rate of atrophy.^23^ In this study, the main stimulated area of the brain was the left temporoparietal junction (TPJ) with 80% motor threshold. Similarly, a study by Bais et al.^11^ found that left rTMS resulted in stronger network contributions to the auditory sensory motor network in the right temporal gyrus, the left frontal parietal lobe network in the right inferior gyrus, and the default mode network in the left middle frontal gyrus. Furthermore, they showed that the bilateral rTMS was associated with the main inhibitory effect on network contribution. It is possible that accurately positioning rTMS coils to target abnormally activated brain regions has a certain impact on therapeutic efficacy.^11^,^24^

TPJ is a heterogeneous associated cortex composed of heterogeneous subregions with high inter-individual variability.^25^,^26^ The system is not accurate enough to guide the TMS coil to the predefined TPJ subregion.^24^,^27^ Therefore, stimuli may be transmitted to different functional TPJ subregions on different treatment days.^25^-^27^ This imprecise stimulation may not achieve the cumulative effect of relieving AVH symptoms in patients with schizophrenia.^11^,^28^ A placebo-controlled randomized controlled trial (RCT) by Hua et al.^28^ found that imaging-guided rTMS was significantly associated with AVH improvement by accurately targeting abnormal brain network nodes or connections and providing different electric field strengths based on individualized AVH networks. This treatment mode aligns with the concept of personalized and precise treatment.^28^

This study demonstrated that, after the final treatment, the general psychopathological symptoms, positive symptom scores, and PANSS total score in patients treated with rTMS and risperidone were significantly lower than those who received risperidone alone. This finding is consistent with those of Lee et al.^29^ and Sun Y et al.^30^ However, at a one-month follow-up, there was no statistically significant difference in the PANSS scores between the two groups. This may be due to the fact that at one month of follow-up, the rTMS-induced LTD effect has largely subsided, and the excitability of the left temporal gyrus may gradually return to pre-treatment levels. This leads to a weakened inhibitory effect on positive symptoms.^16^,^20^,^31^

In addition to the limitations discussed below, several strengths of this study should be noted. This work contributes additional clinical data on the combined use of low-frequency rTMS and risperidone in patients with AVH, thereby adding evidence from a Chinese patient population where data remain relatively scarce. The strict 1:1 matching of demographic and clinical variables, such as age, gender, BMI, and disease duration, reduced baseline imbalances and enhanced the comparability between groups. Furthermore, the study assessed not only auditory verbal hallucinations but also broader psychopathological domains, including positive, negative, and general symptoms, providing a more comprehensive evaluation of clinical outcomes. The use of multiple validated rating scales (PSYRATS, AHRS, PANSS) also improved the robustness of symptom measurement. Future research should build on these findings by refining rTMS treatment protocols, including adjustments in frequency, intensity, and stimulation targets. The integration of individualized brain mapping techniques, such as fMRI- or EEG-guided neuronavigation, may improve targeting accuracy and therapeutic efficacy. In addition, longer follow-up periods of at least 3–6 months are required to better evaluate the durability of treatment effects. Large-scale, multi-center randomized controlled trials will also be necessary to strengthen the external validity of the findings. Finally, incorporating objective neurophysiological markers such as EEG or P300 alongside clinical scales would provide more rigorous evidence regarding the efficacy of this combined therapeutic approach.

The results of this study indicate that the combination of risperidone and 1-Hz rTMS in the treatment of AVH did not yield any significant benefits in improving auditory hallucination symptoms. Overall, current literature emphasizes that larger-scale studies are needed before reaching clear conclusions about the clinical value of rTMS in treating hallucinations in clinical settings.^13^,^32^ Currently, little is known about the optimal combination between rTMS and antipsychotic drugs. Not all patients respond to rTMS, leaving considerable room for optimizing strategies to enhance clinical efficacy.^32^,^33^

### Limitations:

Firstly, it was a retrospective study based on existing medical records, which inherently restricts reliability and generalizability. Although patients were matched 1:1 on demographic and clinical characteristics such as age, gender, BMI, and disease duration, other potential confounders—including prior treatment resistance and variability in responses to previous antipsychotic therapy—could not be fully controlled. The lack of a randomized controlled trial (RCT) design further limits the ability to establish causal relationships, as potential selection and allocation biases cannot be excluded. Secondly, the relatively small sample size (100 patients, 50 in each group) may have limited the statistical power to detect subtle but clinically relevant differences. Because the sample size was determined by the number of eligible cases during the study period, no a priori power analysis was performed. Future multi-center trials with larger samples and formal power analyses are needed to strengthen statistical validity. Thirdly, treatment adherence could not be uniformly quantified. While risperidone intake was supervised by ward nurses and rTMS sessions were monitored by trained technicians, variability in adherence may still have introduced bias. Similarly, baseline heterogeneity in the severity of auditory verbal hallucinations (AVH) could have influenced treatment outcomes. Prospective studies should adopt standardized adherence-monitoring tools and stratified analyses based on baseline AVH severity to minimize these effects. Fourthly, adverse events were incompletely characterized. Although common reactions such as pain, tachycardia, dizziness, drowsiness, nausea, and vomiting were recorded, detailed information regarding severity, duration, and timing of onset was not consistently available due to the retrospective design. Consequently, the safety profile of the combined therapy could not be comprehensively evaluated. Future research should employ standardized adverse event reporting systems to capture detailed safety data. In addition, outcome evaluation relied exclusively on clinical rating scales (PSYRATS, AHRS, PANSS). While these are validated tools for assessing symptom severity, they are inherently subjective and may introduce bias. Objective neurophysiological measures such as EEG or event-related potentials (e.g., P300) were not available in this study but should be incorporated in future research to provide more robust evidence of treatment efficacy. Finally, both the treatment duration (six weeks) and the follow-up period (one month) were relatively short. Although consistent with prior rTMS studies and clinical feasibility, these timeframes are insufficient to fully assess the long-term or sustained effects of rTMS. This may partly explain why improvements observed at the end of treatment were not maintained at follow-up. Future studies should extend treatment duration and follow-up to at least 3–6 months, optimize stimulation parameters (frequency, intensity, site), and integrate brain-mapping techniques to personalize interventions. Large-scale, prospective, multi-center RCTs will be essential to rigorously validate the efficacy of rTMS combined with risperidone and enhance the generalizability of the findings.

## CONCLUSION

The results of this study suggest that the combination of risperidone and low-frequency rTMS did not produce significant improvements in auditory verbal hallucinations (AVH) in patients with schizophrenia. However, this finding should be interpreted with caution, as the lack of improvement may be influenced by stimulation parameters (e.g., frequency, intensity, site of stimulation) and by individual variations such as age, disease duration, or cortical atrophy. Importantly, the combined therapy was associated with significant improvements in positive symptoms during treatment, indicating potential clinical value beyond AVH. Future research should focus on optimizing rTMS parameters and developing individualized treatment strategies. The use of advanced brain mapping techniques (such as fMRI- or EEG-guided neuronavigation) may improve stimulation accuracy and therapeutic efficacy. Moreover, studies with extended follow-up periods (≥3–6 months) are needed to better evaluate the durability of treatment effects. Larger, multi-center randomized controlled trials will also be essential to validate these findings and enhance their generalizability.

### Authors’ contributions:

**WZ, CC and HC:** Study design. Literature search and manuscript writing.

**WZ, CC, ZH, XY, and XJ:** Data collection, data analysis and interpretation. Critical Review.

**WZ, CC, and HC:** Manuscript revision and validation and is responsible for the integrity of the study.

All authors have read and approved the final manuscript.
